# LPI-SKF: Predicting lncRNA-Protein Interactions Using Similarity Kernel Fusions

**DOI:** 10.3389/fgene.2020.615144

**Published:** 2020-12-09

**Authors:** Yuan-Ke Zhou, Jie Hu, Zi-Ang Shen, Wen-Ya Zhang, Pu-Feng Du

**Affiliations:** College of Intelligence and Computing, Tianjin University, Tianjin, China

**Keywords:** LncRNA-proteins interactions, LncRNA similarities, protein similarities, similarity kernel fusion, laplacian regularized least squares

## Abstract

Long non-coding RNAs (lncRNAs) play an important role in serval biological activities, including transcription, splicing, translation, and some other cellular regulation processes. lncRNAs perform their biological functions by interacting with various proteins. The studies on lncRNA-protein interactions are of great value to the understanding of lncRNA functional mechanisms. In this paper, we proposed a novel model to predict potential lncRNA-protein interactions using the SKF (similarity kernel fusion) and LapRLS (Laplacian regularized least squares) algorithms. We named this method the LPI-SKF. Various similarities of both lncRNAs and proteins were integrated into the LPI-SKF. LPI-SKF can be applied in predicting potential interactions involving novel proteins or lncRNAs. We obtained an AUROC (area under receiver operating curve) of 0.909 in a 5-fold cross-validation, which outperforms other state-of-the-art methods. A total of 19 out of the top 20 ranked interaction predictions were verified by existing data, which implied that the LPI-SKF had great potential in discovering unknown lncRNA-protein interactions accurately. All data and codes of this work can be downloaded from a GitHub repository (https://github.com/zyk2118216069/LPI-SKF).

## Introduction

The human genome is comprised of ~3.2 billion nucleotides, which harbors ~20,000–25,000 protein-coding genes (International Human Genome Sequencing Consortium, 2004). The remaining non-coding genes were once considered to be “junk DNA” in the 1970s due to their weak coding capacity. This included pseudogenes, and simple repeats. (Comings, 1972; Ohno and Smith, 1972). Nonetheless, non-coding sequences have received continuous attention since the 1970s. With the development of sequencing technologies, various ncRNAs (non-coding RNAs), like H19 and XIST (Brannan et al., 1990; Brockdorff et al., 1992; Kung et al., 2013), were discovered in biological regulation processes. lncRNA (long non-coding RNA) is an important type of ncRNAs with a length longer than 200 nt (Mercer et al., 2009; Ma et al., 2013). lncRNAs play important roles in various biological processes (Clark and Mattick, 2011), including transcription (Martianov et al., 2007), splicing (Rintala-Maki and Sutherland, 2009), translation (Beltran et al., 2008), imprinting (Bartolomei et al., 1991), apoptosis (Reeves et al., 2007), and many more. lncRNAs perform their molecular functions by interacting with proteins (Hentze et al., 2018). For example, *MALAT1*, a functional lncRNA, which is highly expressed in several tumors, can bind the tumor suppressor gene *SFPQ* (also known as *PSF*) to release proto-oncogene *PTBP2* (also known as *PTB*) from the *SFPQ*/*PTBP2* complex (Meissner et al., 2000; Tseng et al., 2009; Gutschner et al., 2013; Ji et al., 2014). Studying lncRNA-protein interactions is of great value in understanding the functional mechanism of lncRNAs. However, wet experiments to determining lncRNA-protein interactions are always costly and time-consuming. Therefore, it is crucial to develop efficient and accurate computational methods to predict potential lncRNA-protein interactions.

Recently, a number of computational methods have been developed to predict novel lncRNA-protein interactions. Generally, these methods fall into two categories, the supervised binary classification-based methods and semi-supervised learning-based methods. The most significant difference between these two categories is whether the non-interacting lncRNA-protein pairs are regarded as negative samples or unlabeled samples.

In the binary classification methods, the non-interacting lncRNA-protein pairs are regarded as negative instances. Muppirala et al. encoded RNA-protein pairs using sequence information and trained the model *RPISeq*, using SVM (support vector machine) and RF (random forest) classifiers (Muppirala et al., 2011). By encoding RNA-protein pairs in different ways, two more models were built by SVM or RF classifiers in the following years (Suresh et al., 2015; Xiao et al., 2017). Wang et al. applied a novel extended naive-Bayes classifier on sequence-based features to predict potential protein-RNA interactions (Wang et al., 2013). Ensemble learning was widely applied in combining various machine learning algorithms in predicting lncRNA-protein interactions (Deng et al., 2018; Hu et al., 2018; Wekesa et al., 2019). Despite all these efforts, selecting veracious negative instances is still the most challenging problem in training binary classification-based models. Moreover, the dataset in predicting lncRNA-protein interactions is always highly imbalanced in nature, which could influence the prediction performances in many ways.

In the semi-supervised learning methods, non-interacting lncRNA-protein pairs were considered as unlabeled instances. Lu et al. introduced the matrix multiplication method to score each potential protein-RNA pair (Lu et al., 2013). Li et al. (2015) utilized the RWR (random walk with restart) algorithm on the lncRNA-protein-protein heterogeneous network to predict lncRNA-protein interactions. Serval prediction models were established by the MF (matrix factorization) algorithm, which separates the adjacency matrix into two talent feature vectors (Liu et al., 2017; Ma et al., 2019; Zhang T. et al., 2020). Zhao et al. integrated the RWR and MF algorithm to construct a prediction model (Zhao et al., 2018). A label propagation algorithm is another common recommendation algorithm, two models were built based on label propagation algorithms (Zhang et al., 2018a; Zhu et al., 2019). Meanwhile, some other machine learning algorithms were also adapted in the prediction of lncRNA-protein interactions, including feature projection ensemble learning (Zhang et al., 2018b), KATZ scoring schemes (Zhang et al., 2019), the kernel ridge regression algorithm (Shen et al., 2019), and the depth-first search algorithm (Zhang H. et al., 2020).

Although existing computational models have achieved great performances, there are still some problems that should be solved. With the development of high-throughput sequencing technology, a large number of novel lncRNAs have been discovered. Unlike lncRNAs, that were deposited in the database long ago, little is known about the interacting proteins of these newly identified lncRNAs. Therefore, few existing models can infer potential interacting proteins for these lncRNAs (Zhang et al., 2018b; Zhang T. et al., 2020).

In this paper, we proposed a new model to predict *l*ncRNA-*p*rotein interactions based on the similarity kernel fusion approach, namely LPI-SKF. Multiple similarities between lncRNAs and proteins were first calculated. These similarities were integrated to obtain a comprehensive similarity. Ultimately, the Laplacian regularized least squares framework was applied to build the predictive model. Five-fold cross-validation was used to estimate the performance of LPI-SKF in this work. The LPI-SKF achieved an AUROC (area under receiver operating characteristics curve) of 0.909 and an AUPR (area under precision-recall curve) of 0.685, which indicated that the LPI-SKF method could identify unknown lncRNA-protein interactions accurately. Moreover, LPI-SKF could also be used to identify interacting partners for novel lncRNA/proteins. A total of 19 out of our 20 top-ranked lncRNA-protein interaction predictions were confirmed by existing data.

## Materials and Methods

**Figure 1:**
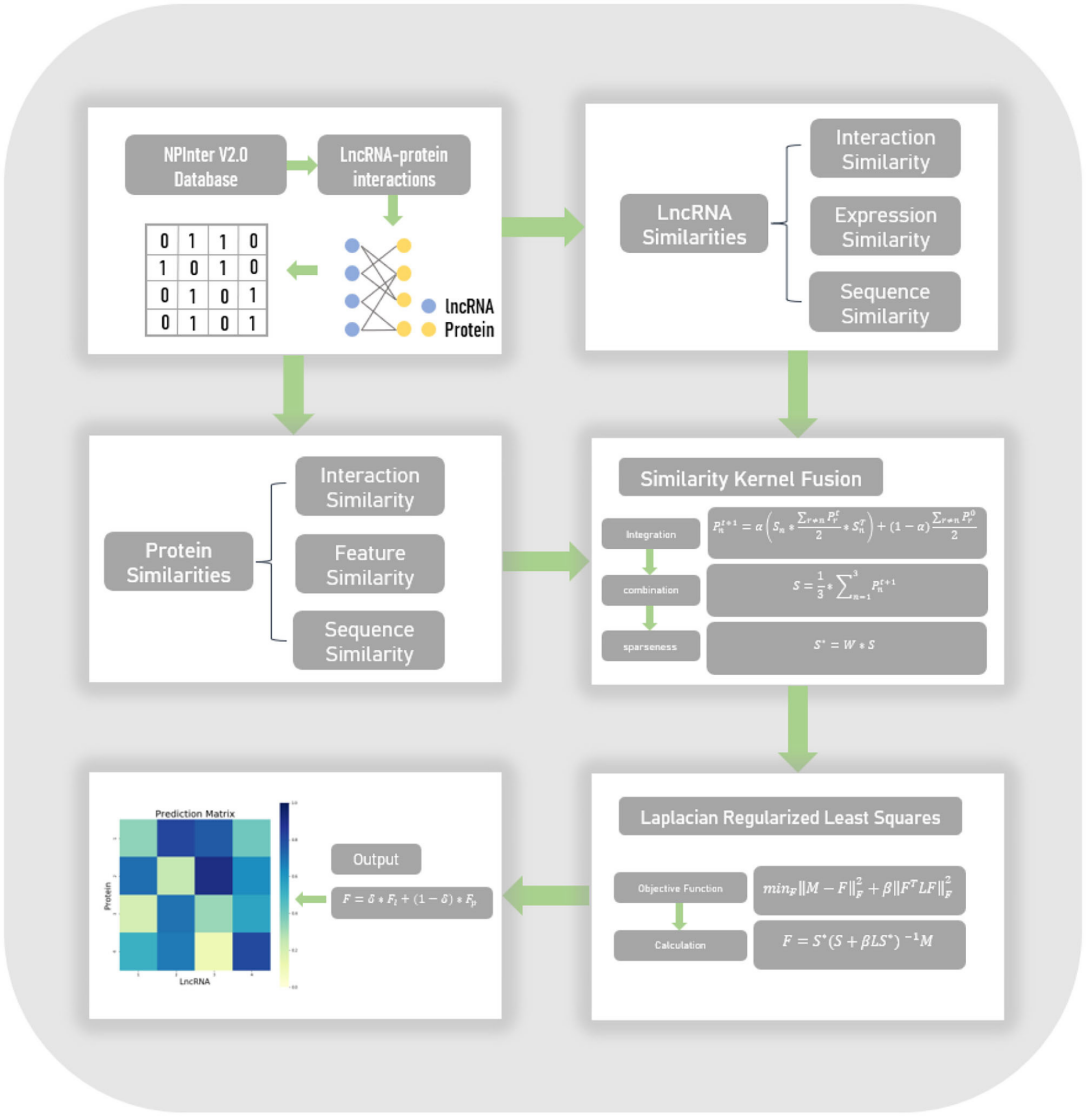
The flowchart of the entire work. Known lncRNA-protein interactions were downloaded from the NPInter V2.0 database to form a heterogeneous network. Three different similarities of both lncRNAs and proteins were calculated, subsequently. Afterward, the similarity kernel fusion (SKF) approach was utilized to integrate these similarities. Finally, the Laplacian regularized least squares (LapRLS) framework was used to build the prediction model.

In this work, we proposed an lncRNA-protein interaction prediction model, named LPI-SKF. This model can be summarized in four steps, which are shown in Figure 1. Firstly, we collected experimentally verified lncRNA-protein interactions in the NPInter V2.0 database and constructed the heterogeneous network. Secondly, based on the assumption that similar lncRNAs tend to interact with similar proteins and vice versa, we calculated three different pairwise similarities for lncRNAs, and three different pairwise similarities for proteins, respectively. Thirdly, to synthesize the similarity information in different aspects and to also reduce noise, the SKF approach was utilized to integrate the lncRNA similarities and protein similarities. Finally, considering the network structure information, we combined the Laplacian regularization and the least squares method to build our prediction model.

### Dataset Curations

NPInter is an integrated database of ncRNA interactions, which includes vast interactions between ncRNAs and biomolecules uncovered by various high-throughput sequencing approaches (Yuan et al., 2014). lncRNA-protein interactions collected in NPInter have been utilized as materials in numerous related studies. For a better comparison, we collected lncRNA-protein interactions from the NPInter V2.0 database according to the previous study (Zhang et al., 2018a). Ultimately, 4158 lncRNA-protein interactions including 990 lncRNAs and 27 proteins were obtained. Afterward, the sequences and expressions of lncRNAs and the sequences of proteins Were downloaded from the NONCODE database and the SUPERFAMILY database, separately (Fang et al., 2018; Pandurangan et al., 2019).

### Similarities for lncRNAs and Proteins

This work is based on the assumption that similar lncRNAs tend to interact with similar proteins and vice versa. Hence, defining appropriate similarity is of great importance in predicting lncRNA-protein interactions. We employed three different pairwise similarities of lncRNAs, including the interaction similarity, the expression similarity, and the sequence similarity. We also applied three different similarities of proteins, including the interaction similarity, the statistical feature similarity, and the sequence similarity. With all these similarity definitions, we proposed to use the similarity kernel fusion strategy to establish a universal and comprehensive similarity kernel matrix to predict potential lncRNA-protein interactions.

#### The Interaction Profile Similarities

For the convenience of the reader, we first defined the adjacency matrix between lncRNAs and proteins. Let *l*_*i*_ (*i* = 1, 2, …, *n*) be the *i*-th lncRNA, and *p*_*j*_ (*j* = 1, 2, …, *m*) the *j*-th protein. The adjacency matrix **A** can be defined as follows:

(1) $$A={\left\{a_{i,j}\right\}}_{n\times m}=\{\begin{array}{cc} 1 & l_{i}\text{ interacts with }p_{j}\text{,}\\ 0 & \text{Otherwise} \end{array}$$

The interaction profile of the *i*-th lncRNA is the *i*-th row of matrix **A**, which can be noted as the **A**_i*_, while the interaction profile of the *j*-th protein is the *j*-th column of matrix **A**, which can be noted as the **A**_*j*_.

The interaction similarity between *l*_*u*_ and *l*_*v*_ can be defined as:

(2) $$\begin{array}{l} s_{l,0}\left(u,v\right)=\exp\left(-\gamma_{l}{||{\text{A}}_{u^{*}}-{\text{A}}_{v^{*}}||}^{2}\right), \end{array}$$

where

(3) $$\begin{array}{l} \gamma_{l}=n/\overset{n}{\underset{i=1}{\sum }}{||{\text{A}}_{i^{*}}||}^{2}, \end{array}$$

and ||.|| is the 2-norm operator.

Similarly, the interaction similarity between *p*_*u*_ and *p*_*v*_ can be defined as:

(4) $$\begin{array}{l} s_{p,0}\left(u,v\right)=\exp\left(-\gamma_{p}{||{\text{A}}_{u}-{\text{A}}_{v}||}^{2}\right), \end{array}$$

where.

(5) $$\begin{array}{l} \gamma_{p}=m/\overset{m}{\underset{j=1}{\sum }}{||{\text{A}}_{j}||}^{2}. \end{array}$$

#### lncRNA Expression Profile Similarity

The expression profiles of lncRNAs in 24 different tissues can be downloaded from the NONCODE database. The expression profile of the *i*-th lncRNA can be noted as **e**_*i*_. The expression profile similarity is defined as follows:

(6) $$s_{l,1}\left(u,v\right)=\{\begin{array}{cc} \frac{1}{2}\left(1+\rho_{u,v}\right) & u\neq v\\ 0 & u=v \end{array},$$

where ρ_*u*,*v*_ is the Pearson's correlation coefficient between **e**_*u*_ and **e**_*v*_. It can be calculated as follows:

(7) $$\begin{array}{l} \rho_{u,v}=\frac{\mathrm{cov}\left({\text{e}}_{u},{\text{e}}_{v}\right)}{\sigma \left({\text{e}}_{u}\right)\sigma \left({\text{e}}_{v}\right)}, \end{array}$$

where cov() is the covariance, and σ is the standard deviation operator.

#### Protein Pairwise Sequence Alignment Similarity

Blast+ is a local alignment search tool, which was utilized to calculate the alignment score of proteins in this work (Camacho et al., 2009). We used blast+ to align *p*_*u*_ against *p*_*v*_. The bit score in this alignment can be noted as *b*_*u*,*v*_. The pairwise sequence alignment similarity can be defined as:

(8) $$s_{p,1}\left(u,v\right)=\{\begin{array}{cc} b_{u,v}/b_{u,u} & u\neq v\\ 0 & u=v \end{array}$$

It worth noting that *s*_*p*,1_ is not symmetric. Therefore, we have *s*_*p*,1_(*u, v*) ≠ *s*_*p*,1_(*v, u*).

#### Sequence Statistical Feature Similarity

RNA is composed of four types of ribonucleotide (A, G, C, U). According to the previous work, we calculated the percentage of these four nucleotides and 16 dinucleotides (AA, AG, AC, AU, …) to represent each lncRNA in a 20-D vector (Zhang et al., 2018a). We employed CTD (composition-transition-distribution) features (Li et al., 2006) in this work. Twenty different amino acids were divided into three groups, according to their hydrophobicity, normalized van der Waals volume, polarity, and polarizability. Each protein was represented as a 504-D vector. Linear neighborhood similarity (LNS), which is based on the hypothesis that each vector can be represented by their *k*-nearest neighbors, was adopted to compute the similarity between statistical features (Wang and Zhang, 2008; Deng et al., 2020) for lncRNA and proteins, respectively. The sequence statistical feature similarity between *l*_*u*_ and *l*_*v*_ can be noted as *s*_*l*,2_(*u, v*), while the similarity between *p*_*u*_ and *p*_*v*_ can be noted as *s*_*p*,2_(*u, v*).

### Similarity Kernel Fusion

Three different lncRNA similarities (*s*_*l*__*q*_ *q* = 0, 1, 2) and three different protein similarities (*s*_*p*,*q*_ *q* = 0, 1, 2) were calculated in the above sections. Furthermore, the similarity kernel fusion (SKF) algorithm was utilized to integrate these similarities and obtain a more comprehensive similarity.

We take the similarities of lncRNA as an example. Firstly, we can normalize the three lncRNA similarities (*s*_*l*,*q*_ *q* = 0, 1, 2) as follows:

(9) $$\begin{array}{l} \theta_{l,q}\left(u,v\right)=\frac{s_{l,q}\left(u,v\right)}{\overset{n}{\underset{t=1}{\sum }}s_{l,q}\left(t,v\right)}, \end{array}$$

where θ_*l*,*q*_ is the normalized similarity corresponding to *s*_*l*,*q*_. The matrix composed by the normalized similarity is noted as:

(10) $$\begin{array}{l} \Theta_{l,q}={\left\{\theta_{l,q}\left(u,v\right)\right\}}_{n\times n}. \end{array}$$

Secondly, we created a neighbor-constrained normalization for each lncRNA similarity. Given *l*_*u*_ and *s*_*l*,*q*_, we collected the *k* most similar lncRNA as a set *N*_*l*,*q*_(*u, k*). The neighborhood constrained normalization of the *s*_*l*,*q*_ can be defined as follows:

(11) $$\begin{array}{l} \varphi_{l,q}\left(u,v\right)=\frac{s_{l,q}\left(u,v\right)I_{l,q,k}\left(u,v\right)}{\overset{n}{\underset{t=1}{\sum }}s_{l,q}\left(u,t\right)I_{l,q,k}\left(u,t\right)}, \end{array}$$

where

(12) $$I_{l,q,k}\left(u,v\right)=\{\begin{array}{cc} 1 & l_{v}\in N_{l,q}\left(u,k\right)\\ 0 & l_{v}\notin N_{l,q}\left(u,k\right) \end{array}$$

The matrix composed by the neighborhood constrained normalization is noted as:

(13) $$\begin{array}{l} \Phi_{l,q}={\left\{\varphi_{l,q}\left(u,v\right)\right\}}_{n\times n}. \end{array}$$

The three similarity matrices were integrated using the following iterative process:

(14) $$\begin{array}{l} \Theta_{l,q}\left(\lambda +1\right)=\frac{1}{2}\alpha \left(\Phi_{l,q}\underset{r\neq q}{\sum }\Theta_{l,r}\left(\lambda \right)\Phi_{l,q}^{T}\right)\\ \;+\frac{1}{2}\left(1-\alpha \right)\underset{r\neq q}{\sum }\Theta_{l,r}\left(0\right), \end{array}$$

where α is a weight coefficient between 0 and 1, *T* is the transpose operator in matrix algebra, λ is the iterative round parameter, and

(15) $$\begin{array}{l} \Theta_{l,r}\left(0\right)=\Theta_{l,r}. \end{array}$$

After *z* rounds of the iterative process, we obtained the final integration similarity matrix as

(16) $$\begin{array}{l} \Theta_{l}=\frac{1}{3}\left(\Theta_{l,0}\left(z\right)+\Theta_{l,1}\left(z\right)+\Theta_{l,2}\left(z\right)\right) \end{array}$$

Although more information is retained in the similarity fusion, more noise is apparent simultaneously. By considering the *k* most similar lncRNAs of each lncRNA, we defined an indicator function as follows:

(17) $$w_{l,k}\left(u,v\right)=\{\begin{array}{cc} 1 & I_{l,0,k}\left(u,v\right)=I_{l,1,k}\left(u,v\right)=I_{l,2,k}\left(u,v\right)=1\\ 0 & I_{l,0,k}\left(u,v\right)=I_{l,1,k}\left(u,v\right)=I_{l,2,k}\left(u,v\right)=0\\ 0.5 & otherwise \end{array}$$

The final adjusted lncRNA similarity is defined as follows:

(18) $$\begin{array}{l} {\text{S}}_{l,k}={\left\{\theta_{l}\left(u,v\right)w_{l,k}\left(u,v\right)\right\}}_{n\times n}, \end{array}$$

where θ_*l*_(*u, v*) is the element in the *u*-th row and the *v*-th column of the matrix **Θ**_*l*_.

By applying protein similarities, and using Eqs. (9)–(18), we obtained the adjusted protein similarity matrix **S**_p,*k*_. The value of *k* in computing protein similarities is not necessarily the same as that of the lncRNAs.

### Laplacian Regularized Least Squares

In this work, Laplacian regularized least squares (LapRLS) were utilized to construct the prediction model. Since we obtained the lncRNA similarity matrix and the protein similarity matrix, we could estimate the lncRNA-protein interactions from either the lncRNA similarity matrix or the protein similarity matrix. Without losing generality, we took the lncRNA similarity matrix as an example.

Let **L**_*l*_ be the Laplacian normalized similarity matrix, which can be defined as follows:

(19) $$\begin{array}{l} {\text{L}}_{l}={\text{D}}_{l}^{-1/2}\left(\text{D}-{\text{S}}_{l,k}\right){\text{D}}_{l}^{-1/2}, \end{array}$$

where **D** is the diagonal matrix of the matrix **S**_l,*k*_.

We then found the estimation of the adjacency matrix by minimizing the following objective function:

(20) $$\begin{array}{l} \underset{F_{l}}{\min}||\text{A}-{\text{F}}_{l}{||}_{F}^{2}+\beta_{l}||{\text{F}}_{l}^{T}{\text{L}}_{l}{\text{F}}_{l}{||}_{F}^{2}, \end{array}$$

where **A** is the adjacency matrix, **F**_*l*_ is the prediction matrix from lncRNA similarities, β_*l*_ is a weighting parameter, and ||.||_*F*_ is the F-norm operator.

We obtained the prediction matrix from lncRNA similarities by calculating the derivative of the objective function as follows:

(21) $$\begin{array}{l} {\text{F}}_{l}={\text{S}}_{l,k}{\left({\text{S}}_{l,k}+\beta_{l}{\text{L}}_{l}{\text{S}}_{l,k}\right)}^{-1}\text{A} \end{array}$$

Similarly, we applied Eqs. (19)–(21) on protein similarities to obtain the prediction matrix from protein similarities, as follows:

(22) $$\begin{array}{l} {\text{F}}_{p}={\text{S}}_{p,k}{\left({\text{S}}_{p,k}+\beta_{p}{\text{L}}_{p}{\text{S}}_{p,k}\right)}^{-1}\text{A}. \end{array}$$

Finally, we integrated the above two prediction matrixes to obtain our final prediction matrix, as follows:

(23) $$\begin{array}{l} \text{F}=\delta {\text{F}}_{l}+\left(1-\delta \right){\text{F}}_{p}, \end{array}$$

where δ ε (0, 1) is a weighting coefficient.

### Performance Estimation Protocol

The prediction performances of the LPI-SKF method was estimated using 5-fold cross-validations. We applied the AUROC and the AUPR as the main performance indicators. We also applied three performance statistics, including precision (*pre*), recall (*rec*), and the F1-score (*f*), which can be calculated as follows:

(24) $$\begin{array}{l} pre=\frac{TP}{TP+FP}, \end{array}$$

(25) $$\begin{array}{l} rec=\frac{TP}{TP+FN},\text{and} \end{array}$$

(26) $$\begin{array}{l} f=\frac{2pre\cdot rec}{pre+rec}, \end{array}$$

where *TP, TN, FP*, and *FN* represent the number of true positives, true negatives, false positives, and false negatives, respectively.

For predicting potential lncRNA-protein interactions, all interactions in the adjacency matrix were divided randomly into five parts. Four parts were utilized as the training dataset, while the remaining part was used as the testing dataset. Through five rounds of cross-validation, we obtained the interacting score of every interaction.

As for predicting potential proteins for new lncRNAs, all lncRNAs were split into five groups. Four groups were treated as the training set and the remaining one as the testing set, which was the same as the prediction for new proteins.

### Parameter Calibrations

The primary parts in LPI-SKF are SKF and LapRLS. There are three parameters in the SKF, which are the iteration times *z*, the number of neighbors *k*, and the weighting coefficient α. Since SKF was adopted to integrate the lncRNA similarities and the protein similarities separately, we calculated the AUC from lncRNA similarities and protein similarities, respectively to find the optimal α. Since the value range of α is between 0 and 1, we took α within a range of 0.1–0.9 with the step of 0.1 for calculation convenience. The prediction performances were estimated from lncRNAs and proteins separately. As in Table 1, the optimal α for lncRNAs was 0.9, while it was 0.8 for proteins.

**Table 1:** AUC of lncRNA space and protein space with different weighting coefficient α.

**α^a^**	**0.1**	**0.2**	**0.3**	**0.4**	**0.5**	**0.6**	**0.7**	**0.8**	**0.9**
lncRNA^b^	0.898	0.892	0.892	0.893	0.893	0.894	0.894	0.895	0.895
Protein^c^	0.786	0.786	0.787	0.788	0.789	0.796	0.799	0.800	0.799

a *α: the weighting coefficient α in the SKF method*.

b *LncRNA: the performance of LPI-SKF in the lncRNA subspace, AUC was selected as the evaluation index in this part*.

c *Protein: the performance of LPI-SKF in the protein subspace, AUC was selected as the evaluation index in this part*.

**Figure 2:**
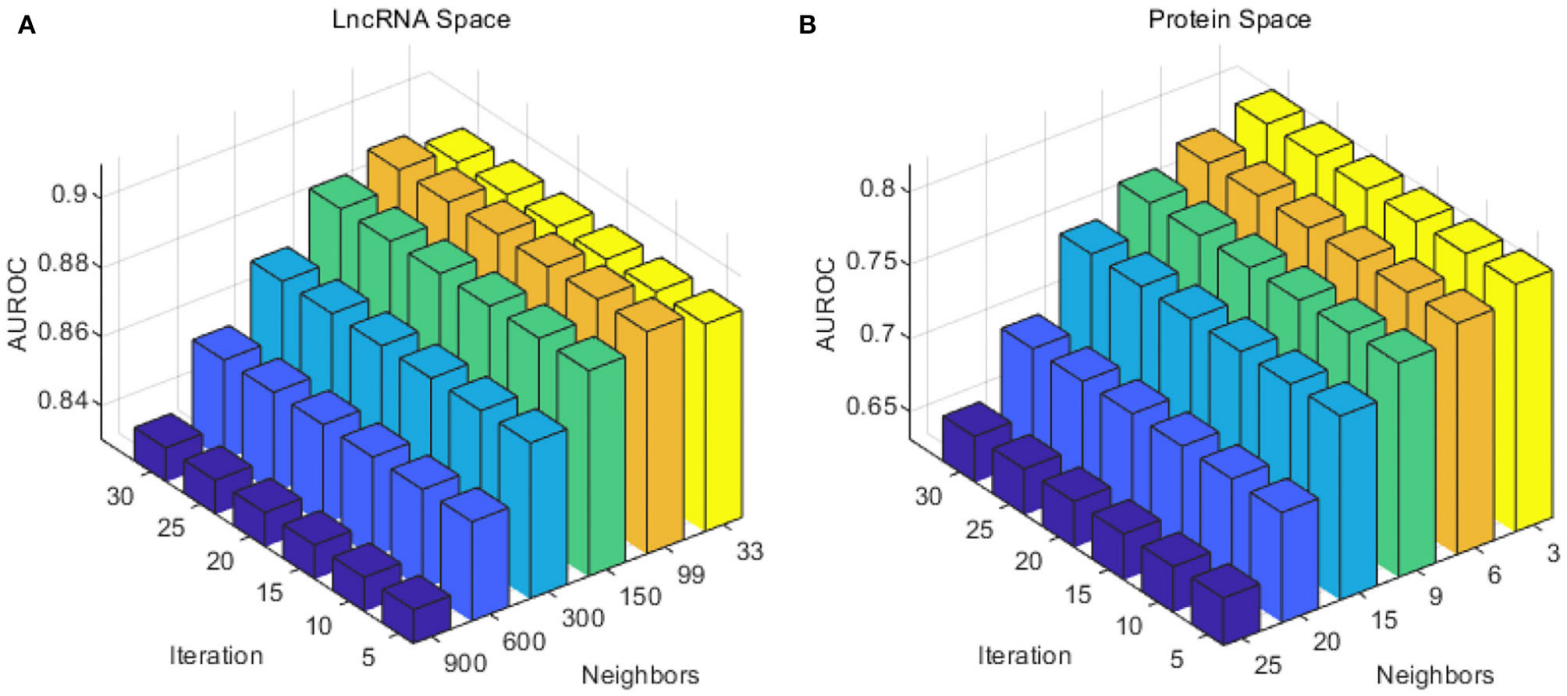
Performance of the combination of different iteration times *T* and neighbor number *k* in the SKF method. **(A)** The AUC of different parameters in the lncRNA subspace. **(B)** The AUC of different parameters in the protein subspace.

Considering the number of lncRNAs and proteins in our work (990 lncRNAs and 27 proteins), the number of neighbors *k* for lncRNA was selected from {33, 99, 150, 300, 600, 900}, and the number of neighbors *k* for proteins from {3, 6, 9, 15, 20, 25}. To reduce calculating time and to test as much as possible, the iteration times *z* was taken from 5 to 30 with a step of 5. As in Figure 2, the optimal number of neighbors *k* for lncRNA was 99, and 3 for proteins. The optimal iteration times *z* was set to 5 for lncRNAs and proteins.

**Figure 3:**
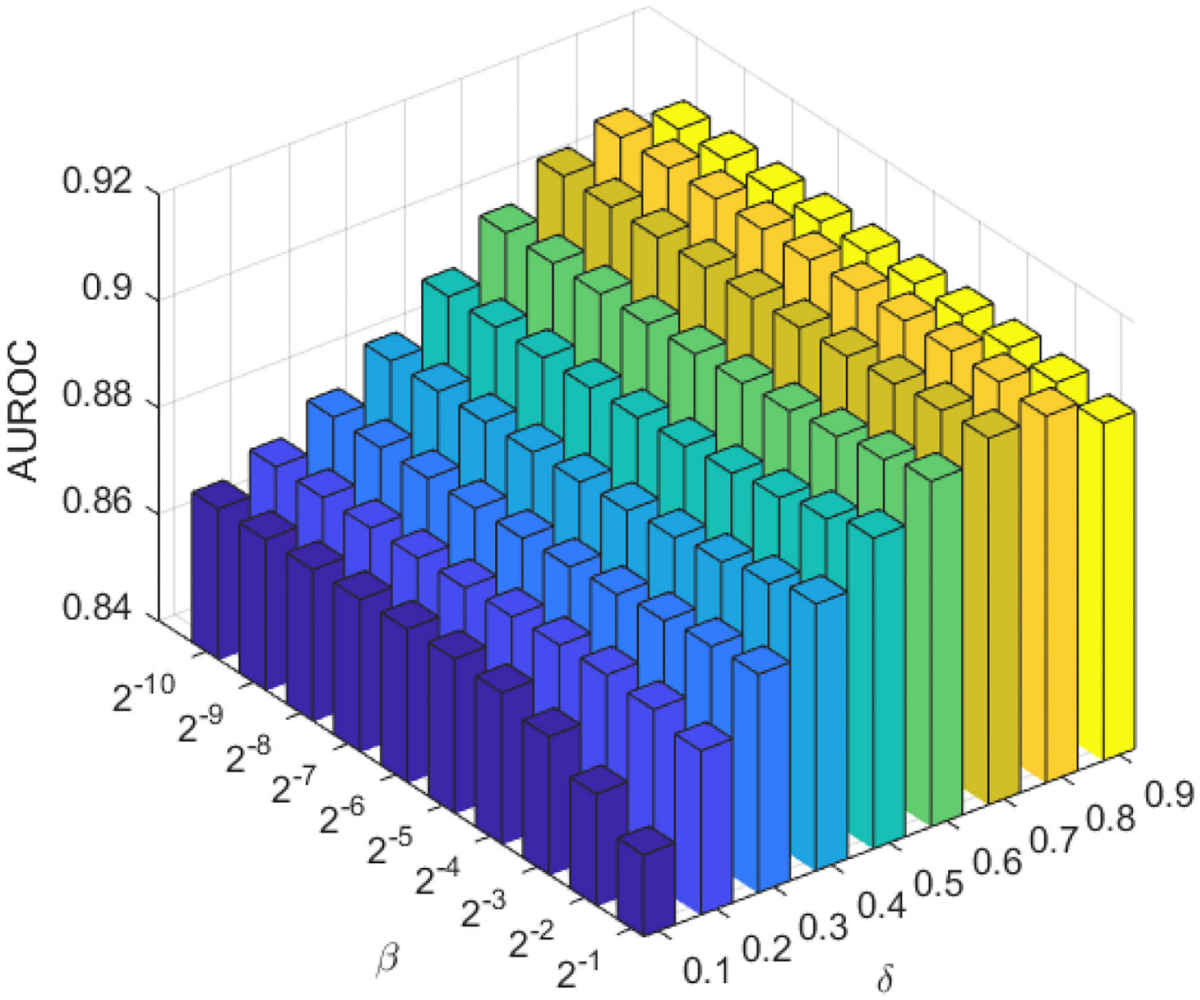
The AUC of the combination of different proportional coefficient β and weighting factor δ in the LapRLS framework.

The weighting parameter β_*l*_ and β_*p*_ are the most important regularization terms in the LapRLS, which can influence the performance directly. In this work, we made β_*l*_ equal to β_*p*_ for convenience. To obtain the optimal performance, we searched β_*l*_ and β_*p*_ both from 2^−10^ to 2^−1^ according to a previous work (Jiang et al., 2018). Since the amount of lncRNAs is much more than proteins, we made δ range from 0.1 to 0.9 with a step of 0.1. As in Figure 3, we chose β_*l*_ = β_*p*_ = 2^−3^, and δ = 0.8.

## Results

### Comparison With Single Similarity

**Figure 4:**
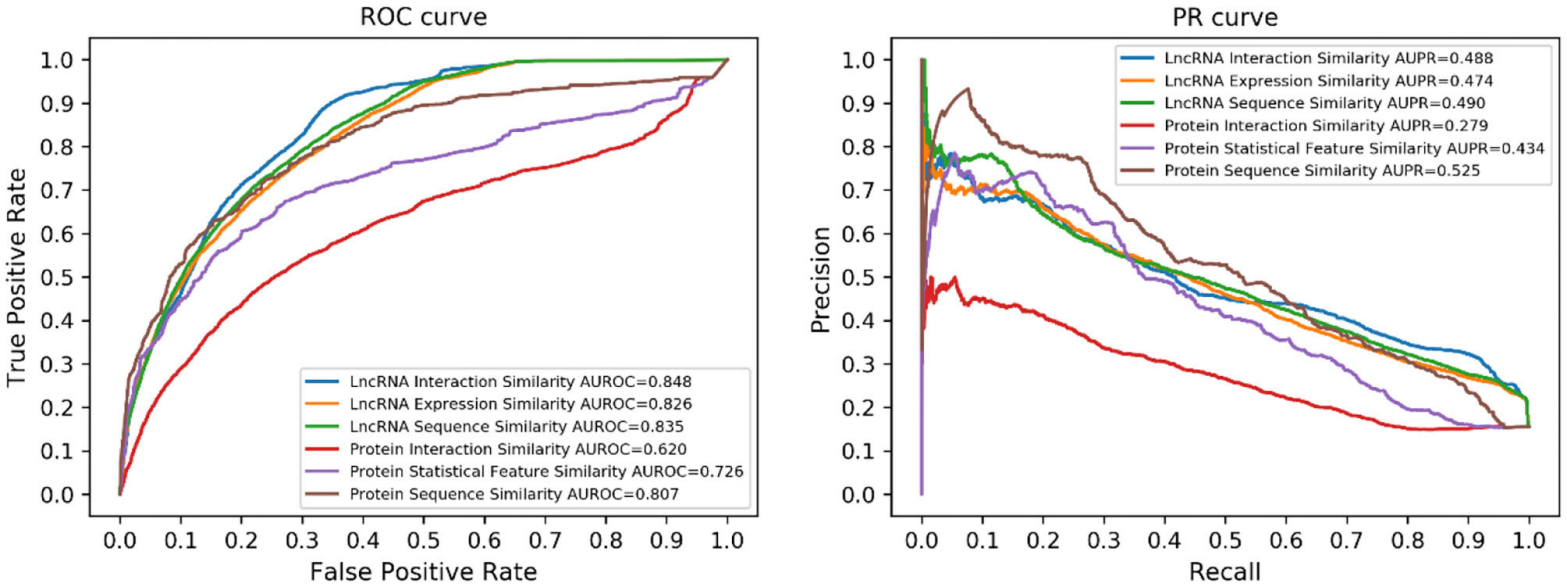
Performance of the single similarity. Six different similarities (three lncRNA similarities and three protein similarities) were utilized in this work, each of them corresponds to a prediction model.

Different types of similarities between both lncRNAs and proteins have been utilized in this work. To demonstrate the benefit of similarity integration, we tested the prediction performance of every single similarity. The results are illustrated in Figure 4. Considering the different numbers of lncRNAs and proteins, performance using lncRNA similarities was better than protein similarities.

### Comparison With Other Fusion Methods

**Figure 5:**
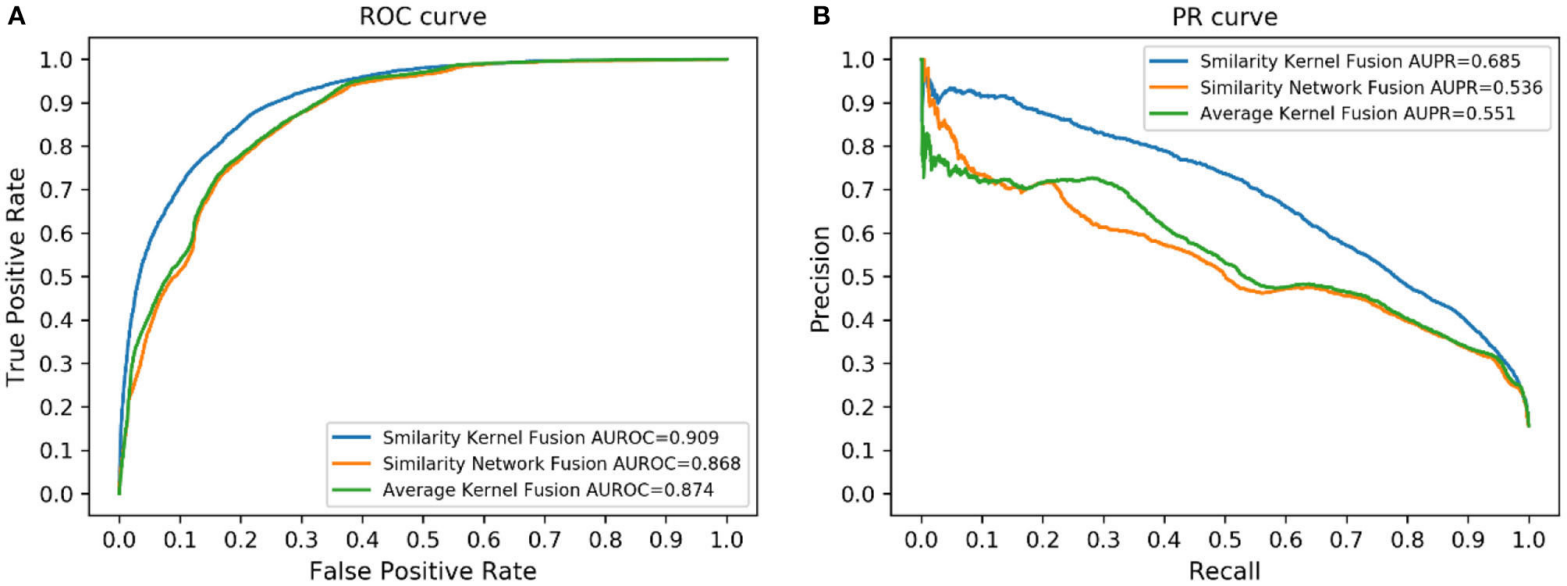
Performance of different similarity fusion methods. **(A)** ROC curves. **(B)** PR curves.

Similarity kernel fusion (SKF) was applied in our study to integrate different similarities, which could integrate similarity information in different aspects and reduce noise. In this part, we compared SKF with another two similarity fusion methods, similarity network fusion (SNF) (Wang et al., 2014) and average kernel fusion (AVG). The results are shown in Figure 5. The results indicated that SKF outperformed the other two methods.

### Comparison With State-Of-the-Art Methods

#### Prediction for Uncovered Interactions

**Figure 6:**
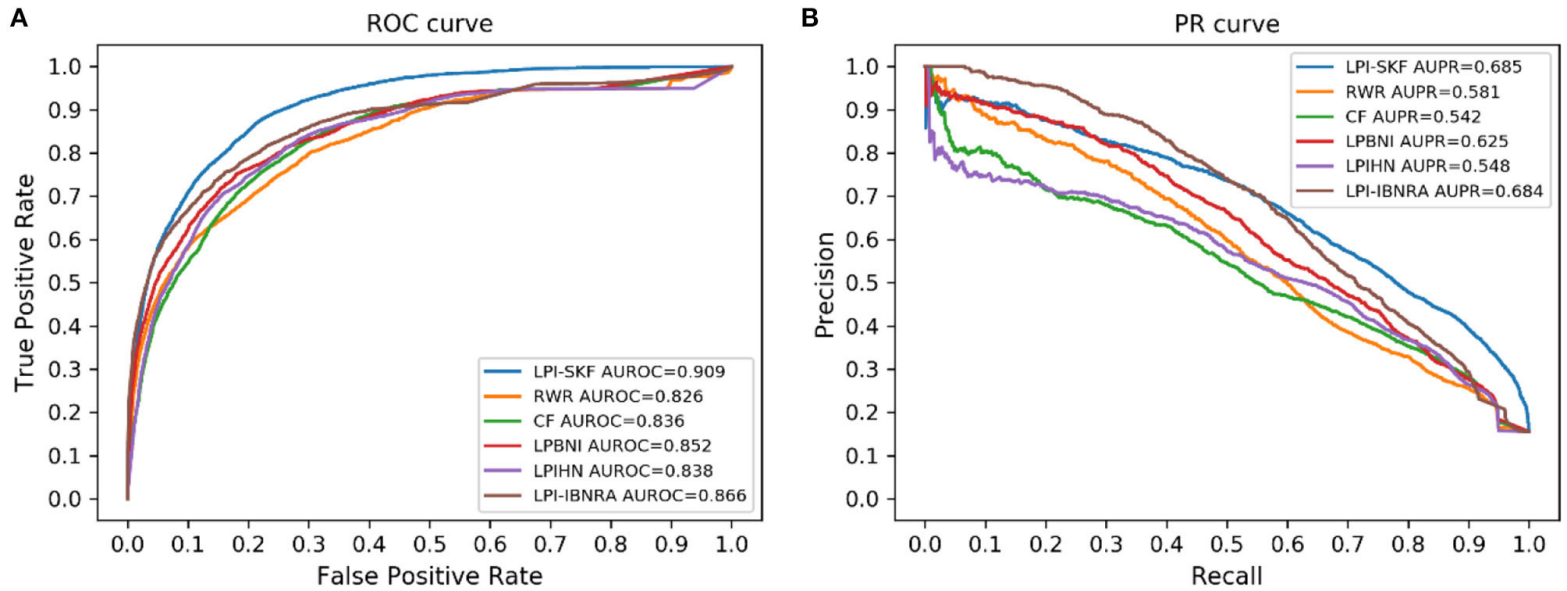
Performance of different methods on the same benchmark dataset. **(A)** ROC curves **(B)** PR curves.

In our study, we compared LPI-SKF with two popular algorithms, RWR (random walk with restart) and CF (collaborative filtering), and three other methods, including LPIHN (Li et al., 2015), LPBNI (Ge et al., 2016), and LPI-IBNRA (Xie et al., 2019). We built six prediction models based on the same benchmarking dataset. Subsequently, the 5-fold cross-validation (5-fold CV) was applied for the comparison. The result is shown in Figure 6. Meanwhile, we selected the threshold value of six models based on the optimal F1-score. Furthermore, the recall, precision, and F1-score under the threshold value were computed to compare these models in other aspects. For a better comparison, the results of the six models are collected in Table 2. From the table, we can see that both the AUC and AUPR of LPI-SKF were higher than the other models.

**Table 2:** Comparison with state-of-the-art prediction methods.

**Methods**	**AUC**	**AUPR**	**Recall**	**Precision**	**F1-Score**
LPI-SKF^a^	0.909	0.685	0.623	0.643	0.633
RWR	0.826	0.581	0.566	0.535	0.550
CF	0.836	0.542	0.633	0.459	0.532
LPBNI	0.852	0.625	0.634	0.533	0.579
LPIHN	0.838	0.548	0.648	0.494	0.560
LPI-IBNRA	0.866	0.684	0.599	0.652	0.624

a *LPI-SKF: the performance of LPI-SKF in the NPInter V2.0 database, the same as the other models*.

Specifically, for the AUC, LPI-SKF received an AUC of 0.909, which increased by 10.05, 8.73, 6.69, 8.47, and 4.72%, respectively, compared with RWR's 0.826, CF's 0.836, LPBNI's 0.852, LPIHN's 0.866, and LPI-IBNRA's 0.864. As for another important index: AUPR, LPI-SKF obtained an AUPR of 0.685, which was higher than all other models, RWR's 0.581, CF's 0.542, LPBNI's 0.625, LPIHN's 0.548, and LPI-IBNRA's 0.684. Meanwhile, the best F1-score of LPI-SKF was also higher than the other models. All these evaluation indexes demonstrate that LPI-SKF outperformed the other state-of-the-art methods.

#### Prediction for Novel lncRNAs/Proteins

**Figure 7:**
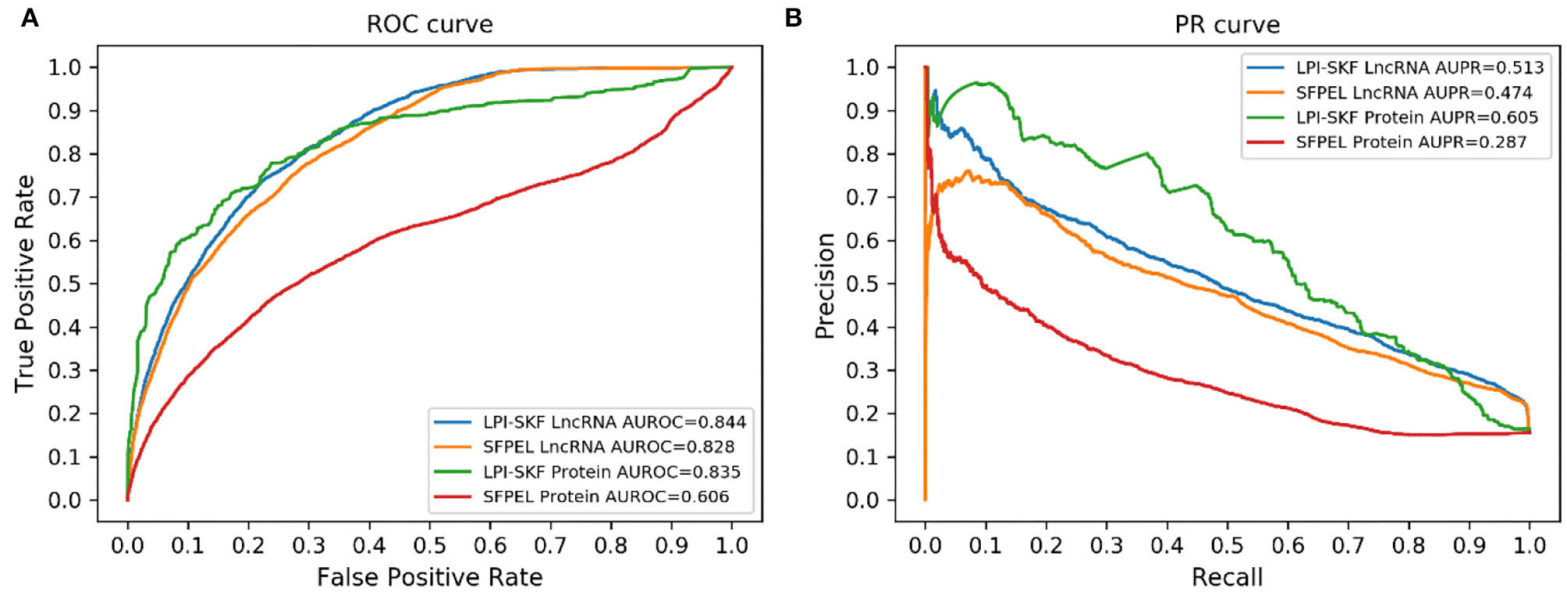
Comparison of the prediction for new lncRNAs/proteins on the same benchmark dataset. **(A)** ROC curves. **(B)** PR curves.

While our model can predict potential interacting lncRNAs/proteins for novel proteins/lncRNAs, we also made a comparison for the prediction of new lncRNAs/proteins. As few methods could predict interacting lncRNAs/proteins for novel proteins/lncRNAs, SFPEL-LPI (Zhang et al., 2018b) was selected for the comparison. Subsequently, we evaluated the performance of the two models in new lncRNAs and new proteins prediction, respectively. The result is shown in Figure 7. For a better comparison, the AUC, AUPR, recall, precision, and F1-score of the two models are shown in Table 3. LPI-SKF obtained an AUC of 0.844 and 0.835 in the prediction of new lncRNAs and proteins, respectively. Comparing with SFPEL, LPI-SKF achieved an AUC improvement of 0.016 and 0.229 in new lncRNAs and proteins prediction, separately.

**Table 3:** Comparison of the prediction for new lncRNAs/proteins.

**Methods**	**AUC**	**AUPR**	**Recall**	**Precision**	**F1-score**
LPI-SKF lncRNA^a^	0.844	0.513	0.653	0.416	0.508
SFPEL lncRNA^b^	0.828	0.474	0.514	0.469	0.490
LPI-SKF protein^c^	0.835	0.605	0.570	0.598	0.584
SFPEL protein^d^	0.606	0.287	0.459	0.266	0.337

a *LPI-SKF lncRNA: prediction performance for the new lncRNAs of LPI-SKF*.

b *SFPEL lncRNA: prediction performance for the new lncRNAs of SFPEL*.

c *LPI-SKF protein: prediction performance for the new proteins of LPI-SKF*.

d *SFPEL protein: prediction performance for the new proteins of SFPEL*.

### Case Studies

To evaluate the prediction effect of LPI-SKF more accurately, we tested the 20 top-ranked interactions in our model based on the NPInter V2.0 database. The result is shown in Table 4. Nineteen of these interactions have been verified in the NPInter V2.0 database, which demonstrates that LPI-SKF performed reputably in actual interaction prediction. Meanwhile, the amount of correctly predicted interactions of the 50 top-ranked interactions, the 100 top-ranked interactions, and the 500 top-ranked interactions are 47, 92, and 458, respectively.

**Table 4:** 20 top-ranked predicted interactions in this work.

**LncRNA^**a**^**	**Species**	**Protein^**b**^**	**Species**	**Confirmed?^**c**^**
NONHSAT130775	Homo sapiens	Q9NUL5	Homo sapiens	Confirmed
NONHSAT137303	Homo sapiens	Q9NUL5	Homo sapiens	Confirmed
NONHSAT118886	Homo sapiens	Q9NUL5	Homo sapiens	Confirmed
NONHSAT035663	Homo sapiens	Q9NUL5	Homo sapiens	Confirmed
NONHSAT124467	Homo sapiens	Q9NUL5	Homo sapiens	Confirmed
NONHSAT010896	Homo sapiens	Q9NUL5	Homo sapiens	Confirmed
NONHSAT092997	Homo sapiens	Q9NUL5	Homo sapiens	None
NONHSAT039675	Homo sapiens	Q9NUL5	Homo sapiens	Confirmed
NONHSAT055307	Homo sapiens	Q9NUL5	Homo sapiens	Confirmed
NONHSAT138539	Homo sapiens	Q9NUL5	Homo sapiens	Confirmed
NONHSAT098625	Homo sapiens	Q9NUL5	Homo sapiens	Confirmed
NONHSAT014009	Homo sapiens	Q9NUL5	Homo sapiens	Confirmed
NONHSAT098480	Homo sapiens	Q9NUL5	Homo sapiens	Confirmed
NONHSAT056108	Homo sapiens	Q9NUL5	Homo sapiens	Confirmed
NONHSAT083698	Homo sapiens	Q9NUL5	Homo sapiens	Confirmed
NONHSAT089678	Homo sapiens	Q9NUL5	Homo sapiens	Confirmed
NONHSAT135851	Homo sapiens	Q9NUL5	Homo sapiens	Confirmed
NONHSAT108616	Homo sapiens	Q9NUL5	Homo sapiens	Confirmed
NONHSAT073620	Homo sapiens	Q9NUL5	Homo sapiens	Confirmed
NONHSAT102823	Homo sapiens	Q9NUL5	Homo sapiens	Confirmed

a *lncRNA: the lncRNA ID in the NONCODE database*.

b *Protein: the protein ID in the UniProt database*.

c *Confirmed?: whether the direct interaction had been confirmed by an experiment in the NPInter V2.0 database*.

## Conclusion

This paper proposed a novel model, named LPI-SKF (lncRNA-protein interactions prediction based on the similarity kernel fusion), to predict potential lncRNA-protein interactions. Serval similarities of both lncRNAs and proteins were integrated to obtain a comprehensive similarity matrix by the SKF method. Furthermore, the LapRLS framework was applied to build the prediction model. Finally, LPI-SKF obtained an AUC of 0.909 and an AUPR of 0.685 in the 5-fold CV framework, which demonstrated that LPI-SKF can infer uncovered lncRNA-protein interactions accurately.

To evaluate the performance of LPI-SKF, serval state-of-the-art methods were compared to LPI-SKF on the same benchmarking dataset. Finally, LPI-SKF received an AUC of 0.909 and an AUPR of 0.685 in the 5-fold cross-validation framework, both higher than the other models. More importantly, LPI-SKF could also predict potential interacting proteins/lncRNAs for novel lncRNAs/proteins precisely. For a better comparison, we also compared LPI-SKF with another model, SFPEL, on the same database and the same random seed. The result showed that LPI-SKF performed much better both in the prediction for new lncRNAs and new proteins.

## Data Availability Statement

Publicly available datasets were analyzed in this study. This data can be found at: https://github.com/zyk2118216069/LPI-SKF.

## Author Contributions

Y-KZ curated the dataset, designed, and implemented the algorithm, performed the experiments, and collected the results. JH, Z-AS, and W-YZ helped in collecting the data, and calibrating the parameters of the algorithm. P-FD directed the whole study, conceptualized the algorithm, analyzed the results, and wrote the manuscript. All authors contributed to the article and approved the submitted version.

## Conflict of Interest

The authors declare that the research was conducted in the absence of any commercial or financial relationships that could be construed as a potential conflict of interest.
